# Intelligent Breast Mass Classification Approach Using Archimedes Optimization Algorithm with Deep Learning on Digital Mammograms

**DOI:** 10.3390/biomimetics8060463

**Published:** 2023-10-01

**Authors:** Mohammed Basheri

**Affiliations:** Information Technology Department, Faculty of Computing and Information Technology, King Abdulaziz University, Jeddah 21589, Saudi Arabia; mbasheri@kau.edu.sa

**Keywords:** bioinspired algorithms, artificial intelligence, computer-aided diagnosis, breast mass, deep learning

## Abstract

Breast cancer (BC) has affected many women around the world. To accomplish the classification and detection of BC, several computer-aided diagnosis (CAD) systems have been introduced for the analysis of mammogram images. This is because analysis by the human radiologist is a complex and time-consuming task. Although CAD systems are used to primarily analyze the disease and offer the best therapy, it is still essential to enhance present CAD systems by integrating novel approaches and technologies in order to provide explicit performances. Presently, deep learning (DL) systems are outperforming promising outcomes in the early detection of BC by creating CAD systems executing convolutional neural networks (CNNs). This article presents an Intelligent Breast Mass Classification Approach using the Archimedes Optimization Algorithm with Deep Learning (BMCA-AOADL) technique on Digital Mammograms. The major aim of the BMCA-AOADL technique is to exploit the DL model with a bio-inspired algorithm for breast mass classification. In the BMCA-AOADL approach, median filtering (MF)-based noise removal and U-Net segmentation take place as a pre-processing step. For feature extraction, the BMCA-AOADL technique utilizes the SqueezeNet model with AOA as a hyperparameter tuning approach. To detect and classify the breast mass, the BMCA-AOADL technique applies a deep belief network (DBN) approach. The simulation value of the BMCA-AOADL system has been studied on the MIAS dataset from the Kaggle repository. The experimental values showcase the significant outcomes of the BMCA-AOADL technique compared to other DL algorithms with a maximum accuracy of 96.48%.

## 1. Introduction

Breast cancer (BC) is a major serious problem which is extremely common worldwide. Early diagnosis and identification perform the main protection from this disease, and these are attained through imaging methods, namely mammography [1]. The diagnostic procedures for BC need medical checkups. Breast mass classification refers to the process of categorizing breast masses or tumors into different classes or categories based on certain characteristics or features. This classification is a critical step in breast cancer diagnosis and plays a crucial role in determining the appropriate treatment and management strategies. Computer-aided diagnosis (CAD) methods depend upon medical screening which can be suitable and which can minimize the specialist’s efforts. CAD analysis plays a considerable part in the diagnosis of breast cancer and can be effectively employed in biomedical science [2]. The widespread CAD-based medical checkups are Magnetic Resonance Imaging (MRI), ultrasound, and mammogram. Mammograms can be a highly efficient, lower-dose, reliable, and less invasive screening method for the early identification of BC [3]. The use of automated CAD methods with mammograms increases the accuracy rate of identification; the operational expedient accelerates the diagnosing process and retains the medical appliances. Additionally, the breast mass shows unique symptoms in BC detection [4]. The marginal data of the breast mass display the growth patterns and biological features. The non-uniform margins of breast mass can usually be malignant cancers, and the accuracy rate of the masses’ segmentation impacts the Malignant Breast Masses Classification (MBMC). Hence, mass classification is an important process utilized in CAD methods to diagnose BC [5], as it supports physicians in treating and analyzing BCs in the earlier phases. Different characteristics exist in breast mass like infection margins, various sizes, and various shapes. Thus, accurate segmentation classification is a common and challenging issue in CAD methods [6].

Digital mammograms often depict difficult and subtle patterns that can be challenging for human radiologists to consistently interpret. Deep learning (DL) models can effectively capture intricate image features and patterns, resulting in significantly enhanced classification accuracy. The incorporation of the DL models into breast mass classification can greatly improve the performance of breast cancer screening programs. Automated systems for BC detection using DL can process a large volume of digital mammograms rapidly, making screening more scalable and accessible. At present, BC detection is challenging because of the variation in breasts in texture, size, location, and shape, and there has been a crucial necessity to decrease false negative and positive conditions and enhance the all-inclusive effectiveness of CAD techniques [7]. With the current development in computers and their improved computational speed and capability, the DL approach is widely recommended in biomedical applications and especially in CAD techniques for mammograms [8]. In recent years, the DL algorithm has made achievements in several computer vision (CV) tasks and has established the ability to address difficult issues in the medical imaging field [9]. Therefore, numerous types of research are proposed to be implemented, specifically in mammography cancer diagnosis, BC segmentation, and classification [10]. Currently, DL approaches have surpassed the basic alteration of convolutional neural network (CNN) methods for existing various complex frameworks, and have also surpassed the image classification outcomes.

This article presents an Intelligent Breast Mass Classification Approach using the Archimedes Optimization Algorithm with Deep Learning (BMCA-AOADL) technique on Digital Mammograms. In the present BMCA-AOADL approach, median filtering (MF)-based noise removal and U-Net segmentation take place as a pre-processing step. For feature extraction, the BMCA-AOADL technique utilizes the SqueezeNet model with AOA as a hyperparameter tuning approach. To detect and classify the breast mass, the BMCA-AOADL technique applies a deep belief network (DBN) approach. The simulation value of the BMCA-AOADL system can be studied on two BC databases.

- An Intelligent BMCA-AOADL technique comprising pre-processing, the SqueezeNet feature extractor, AOA-based hyperparameter tuning, and DBN classification of BC has been developed. To the best of our knowledge, the BMCA-AOADL technique did not exist in the literature.
- Pre-processing steps help to enhance the quality of the digital mammograms and isolate the regions of interest, which are the breast masses.
- The SqueezeNet model is employed for the feature extraction process. The SqueezeNet is known for its efficiency and compact architecture, whilst maintaining a competitive performance, making it a suitable choice for resource-constrained applications like medical image analysis.
- AOA with the SqueezeNet model for feature extraction is designed, where the hyperparameter optimization process using cross-validation helps to boost the predictive outcome of the BMCA-AOADL model for unseen data.
- DBNs are employed for the classification process, which have the capability of modeling complex data distributions, making them suitable for BC image classification tasks.

## 2. Related Works

In [11], a new CAD technique included various phases. The images were segmented in the pre-processing phase and a mask of a lesion was acquired; then, the feature extraction of the DL method was executed using DenseNet-201. Furthermore, hand-crafted features were attained from an image. This technique utilized the CNN framework for extraction, as well as the mutual data selection method, and was supported by the numerous classifiers implemented for selecting the highly sensitive dataset between the features. Malebary and Hashmi [12] introduced a new Breast Mass Classification system termed BMC. This is an enhanced framework that depends on the incorporation of k-mean clustering, the LSTM network of RNN, CNN, and the RF, boosting algorithms for the classification of the breast mass into normal, malignant, and benign.

Jiang et al. [13] presented a three-phase DL approach that depended on an anchor-free object detection technique, called the Probabilistic Anchor Assignment (PAA). Firstly, a single-phase PAA-based detector fully determined suspect BCs in mammography. Secondly, a two-stage ROI detector was developed for more classification and regression of these cancers. Finally, a threshold-adaptive, post-processing method was presented. Eventually, the cancers were categorized using the ROI method. Ragab [14] suggested that a novel deep transfer learning system be developed to accomplish remote sensing scene classification and security. For scene classification, the presented model employed NasNet feature extraction with hyperparameter tuning using a metaheuristic algorithm. The design of the hyperparameter optimization technique helped to adjust the hyperparameter values, which resulted in the achievement of enhanced classification performance. In [15], a DL technique was proposed which included three stages. The pre-processing stage primarily considered normalizing the input images to follow the computation. A novel attention mechanism named the global channel attention method supported the DL architecture for learning inter-channel attention and, therefore, enhanced the segmentation effectiveness of DL frameworks. Narayanan et al. [16] introduced a new DL approach that incorporated the DCNNs and the RF approaches for identifying and classifying BCs. The feature extraction could be conducted using the AlexNet algorithm of the DCNNs and the classifier correctness was improved via the RF method. The images were gathered from the different mammography images of predetermined databases.

In [17], a DL-based convolutional neural network (ConvNet) was introduced to integrate both the mammogram and the medical variation for predicting and classifying breast masses to support the specialist’s decision-making methods. This study was designed to perform an automated and overall representation of breast masses employing the suitable layers in the deep ConvNet technique and by executing data augmentation methods. Chakravarthy et al. [18] used a method of TL to extract the features. The classification efficiency of the weighted KNN (w-KNN) method was enhanced by employing the dragonfly optimizer algorithm (DFOA), crow search optimizer algorithm (CSOA), and PSO as a transformation approach.

In [19], a PSO approach was deployed for selecting important features in the data with ANN for the classification model on a University of California Irvine ML database. In Adebiyi et al.’s study [20], the purpose of this examination was to improve the accuracy of ML approaches for BC analysis. This search executed the ML approaches of RF and SVM with the feature extraction model of linear discriminant analysis (LDA) to the Wisconsin BC (WBC) Database. In [21], PSO was recommended to enhance the efficacy of the classifier with the DT approach on a WBC database.

## 3. The Proposed Model

**Figure 1:**
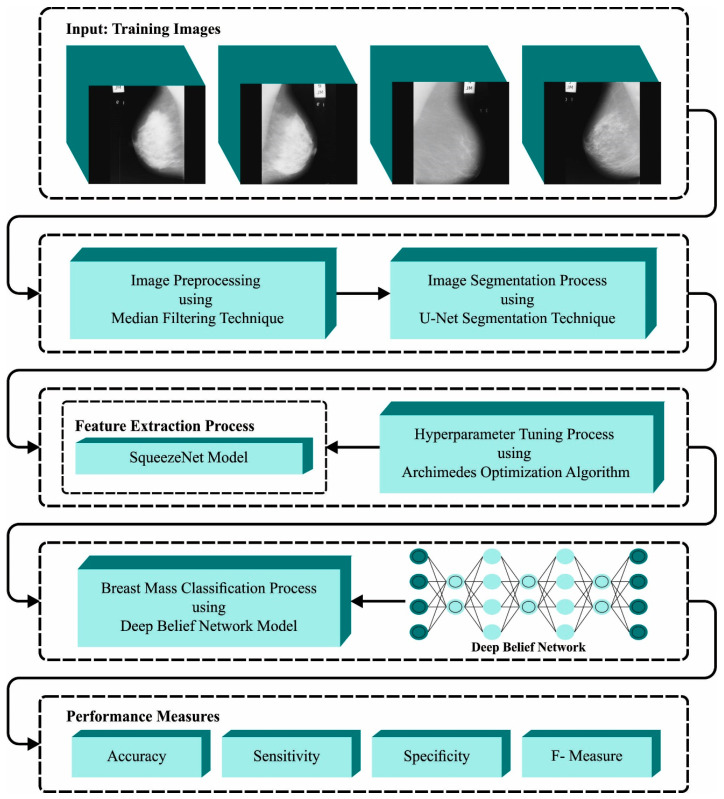
Workflow of BMCA-AOADL algorithm.

In this article, we presented an automated breast mass classification approach named the BMCA-AOADL technique on Digital Mammograms. The main intention of the BMCA-AOADL technique was to exploit the DL model with a bio-inspired algorithm for breast mass classification. In the presented BMCA-AOADL approach, MF-based noise removal, U-Net segmentation, the SqueezeNet feature extractor, AOA-based hyperparameter tuning, and the DBN classification were involved. Figure 1 portrays the workflow of the BMCA-AOADL system.

### 3.1. Image Pre-Processing

In this work, two stages of image pre-processing were involved: MF-based noise filtering and U-Net segmentation. The MF is the filter approach utilized for noise removal in images and signals [22]. It is very critical in the image processing domain as it was established for the maintenance of edges from noise removal. The basic concept of the MF system is to run over the signal entry by entry, exchanging all the entries with the median of neighboring entries. Next, the U-Net segmentation approach was used to segment the affected regions. The U-net is a type of convolutional neural network (CNN) framework that was developed for the segmentation of life science images containing constrained trained data and to categorize the tumors in breast images [23]. The U-Net architecture comprises expansion and contraction. The expansion part is nearly the same as the CNN operation that consists of a convolution layer, and the objective is to frequently apply 3 × 3 convolution filters on the input images. There is a linear activator or ReLU layer after every convolution layer. The ReLU layer employs an activation function to all the neurons, i.e., max(0,x) (which sets the threshold value to 0). Because of the reduction in the number of calculations and parameters within the network, there is a 2 × 2 max-pooling layer with a stride of 2 to decrease the spatial dimension of the image, which causes over-fitting. On the other hand, there is an incremental sampler or in other words, upsampling, together with 2 × 2 convolution layers that is accountable to reduce the particular channel. There is a 3 × 3 convolution layer with a ReLU layer. The objective is to locate the object. During the final layer, there is a 1 × 1 convolutional layer which maps property vectors to a certain class. The steps of the U-Net structure are given below:

(1) Define U-Net structure with the encoding and decoding part;
(2) Input the image to the encoding part and downsample it;
(3) Pass downsampling mapping features to the decoding part;
(4) Upsample the mapping feature and concatenate it with the mapping feature in the respective encoding layer;
(5) Repeat step 4 until image size is attained;
(6) Execute convolution layer to concatenated mapping feature;
(7) Output segmented images.

### 3.2. Feature Extraction Using Optimal SqueezeNet Model

**Figure 2:**
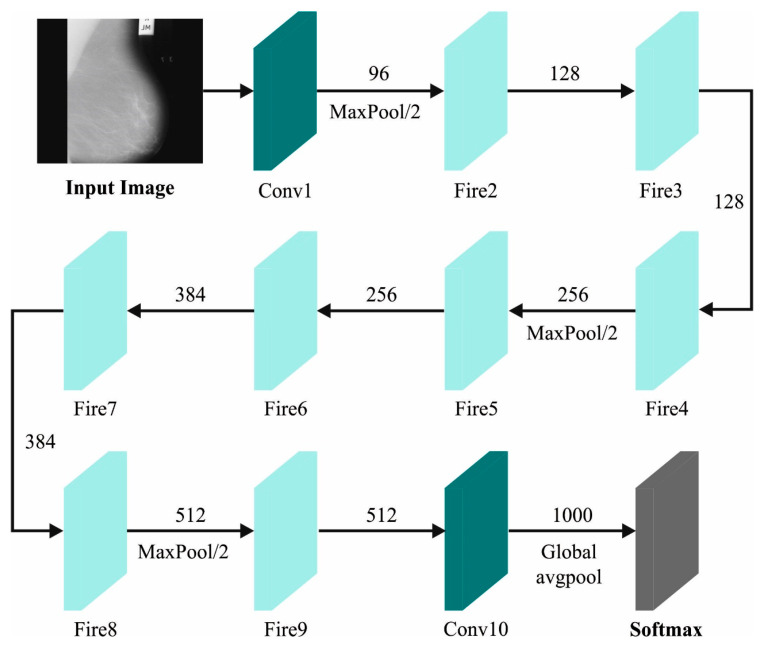
SqueezeNet Architecture.

For feature extraction, the BMCA-AOADL algorithm utilizes the SqueezeNet algorithm. Early DL architectures had the drawback of necessitating considerable amounts of parameters for training [24]. Thus, researcher workers from Deep-scale, University of California, Berkeley, and Stanford University introduced a novel structure named “SqueezeNet” that has a small-scale model and requires fewer training parameters. The SqueezeNet structure includes eight fire modules and one convolution layer. Figure 2 demonstrates the structure of the SqueezeNet.

The fire module has a convolution layer with “n” filters and 1×1 filter size, represented as a “Squeeze” layer. Then, there are two cohesive layers, named “Expand” layers. The initial “Expand” layer comprises 4n filters and  1×1  filter sizes. Likewise, the next “Expand” layer comprises 4n filters and  3×3  filter sizes. These “Squeeze” and “Expand” layers are in sequence. Then, in the first convolutional layer and the second and fourth fire models, a max-pooling layer is utilized (stride = 2), which decreases the feature map size. An FC layer (weight = 1000) is used after the concluding fire module, which transforms the feature maps into a 1D vector. These feature vectors pass over the avgpooling layers. Each prior convolution layer follows a ReLu function. Finally, a softmax layer converts the feature vectors into dual classification.

The hyperparameter tuning of the SqueezeNet approach occurs by employing AOA. AOA was inspired by the rule of Archimedes which is regarded as the law of physics [25]. The Archimedes rules are focused on the object that is completely or partially occupied by the fluid. In AOA, the immersed objects can be supposed to be the population individuals (candidate solutions). This process derives from the initialization of a population with objects; in addition, the position of every object can be initialized from the arbitrary model inside the problem-searching space. Afterwards, the equivalent fitness function (FF) is measured. Iteratively, AOA upgrades the object volumes and densities but its accelerations can upgrade the essential of its collisions with neighbor objects. The initializing method of each object can be executed by exploiting it as:

(1) $$O_{i}=l_{i}+rand\times \left(u_{i}-l_{i}\right),i=1,2,\ldots ,N$$

$u_{i}$ and $l_{i}$ denote the upper as well as lower limits of the $i^{th}$ object and N implies the object counts. The density and volume of all the objects are initialized as:

(2) $$den_{i}=rand,vol_{i}=rand$$

In this example, rand indicates the vector of D-dimension with values within zero and one. The acceleration of all the objects is measured as:

(3) $${acc}_{i}=lb_{i}+rand\times \left(ub_{i}-lb_{i}\right)$$

A main FF can be measured, and an object with better fitness is distributed as $x^{best},$ $den^{best},$ $vol^{best}$, and $acc^{best}$.

The upgrading procedure of the $i^{th}$ object volume and density is executed depending on the following equation:

(4) $$den_{i}^{t+1}=den_{i}^{t}+rand\times \left(den^{best}-den_{i}^{t}\right)$$

(5) $$vol_{i}^{t+1}=vol_{i}^{t}+rand\times \left(vol^{best}-vol_{i}^{t}\right)$$

where t denotes the present iteration and rand implies the random number. Primarily, it is a collision amongst the objects once the object obtains the equilibrium condition. It can be performed as proposed in AOA, utilizing a transfer function that supports a change from the exploration to the exploitation phases.

The formulation of this transfer function is represented as:

(6) $$TF=exp\left(\frac{t-t_{max}}{t_{max}}\right)$$

where $r_{max}$ denotes the maximal iteration counts. Now, the TF value is enhanced slowly with iterations. However, it still accomplishes unity.

(7) $$d^{t+1}=exp\left(\frac{t-t_{max}}{t_{max}}\right)-\left(\frac{t}{t_{max}}\right)$$

The value of $d^{t+1}$ diminishes with time, although the suitable transmission of this variable supports accomplishing exploration and exploitation balances. The exploration step can be defined by collision among the objects; this step can be assumed once the transfer function is 0.5. The acceleration of the $i^{th}$ object at iteration t+1 can be upgraded by choosing random material (mr) as:

(8) $$acc_{i}^{t+1}=\frac{den_{mr}+\iota ƒmol_{mr}\times acc_{mr}}{den_{i}^{t+1}\times vol_{i}^{t+1}}$$

where $vol_{mr}$, $acc_{mr}$, and $en_{mr}$ stand for the volume, acceleration, and density of mr. The exploitation stage from the AOA assumes no collision among the objects; this step can be executed if the value of the transfer function is superior to 0.5. The acceleration of the $i^{th}$ object from the exploitation stage is calculated as:

(9) $$acc_{i}^{t+1}=\frac{den_{best}+vol_{best}\times acc_{best}}{den_{i}^{t+1}\times vol_{i}^{t+1}}$$

where $acc_{best}$, $en_{best},$ and $vol_{best}$ indicate the optimum object acceleration, density, and volume, correspondingly. The normalized acceleration is represented as:

(10) $${acc}_{i-norm}^{t+1}=u\times \left(\frac{acc_{i}^{t+1}-\min\left(acc\right)}{\max\left(acc\right)-\min\left(acc\right)}\right)+l$$

where l and u represent the normalized range; it could be assigned as [0.1,0.9]. Once the object is away from the global optimal, the acceleration values are higher. During case exploration, this step can lead to the exploitation stage being proposed.

The position of the $i^{th}$ particle can be upgraded in the exploration stage by exploiting it as:

(11) $$x_{i}^{t+1}=x_{i}^{t}+C_{1}\times rand\times {acc}_{i-norm}^{t+1}\times d\times \left(x_{rand}-x_{i}^{t}\right)$$

In contrast, the upgrading procedure of particle positions in the exploitation stage is projected as:

(12) $$x_{i}^{t+1}=x_{i}^{t}+F\times C_{2}\times rand\times acc_{i-norm}^{t+1}\times d\times \left(T\times x_{best}-x_{i}^{t}\right)$$

where $C_{1}$ and $C_{2}$ denote the constants represented by the user, T implies the parameter that is based on the transfer function $\left(T=C_{3}\times TF\right),$ $C_{3}$ represents the constant value, $x_{best}$ stands for the best particle position, and F defines the flag deployed for changing the particle motion direction.

(13) $$F=\left\{\begin{array}{l} +1P\leq 0.5\\ -1P>0.5 \end{array}\right.$$

where the value of P is distributed arbitrarily by the user. Finally, the FF can be estimated at the upgrade particle positions after the optimal performance is recorded.

The fitness selection is a crucial factor in the AOA approach. Solution encoding is exploited for assessing the aptitude (goodness) of the candidate solution. Now, the accuracy value is the main condition utilized for designing a fitness function.

(14) $$Fitness=max\left(P\right)$$

(15) $$P=\frac{TP}{TP+FP}$$

where FP and TP signify the false and true positive values.

### 3.3. Image Classification Using the DBN Model

For breast mass classification, the DBN approach can be utilized. The DBN model efficiently leverages the residuals based on the predicted values of the MLP model and the corresponding actual observation to enable prediction [26]. The preliminary step of this component includes the visible layer (VL) of RBM receiving the input datasets—viz., the difference between the observed and predicted values of MLP models. Consequently, these data traverse towards the low hidden layer (HL), where a complex feature extraction system unfolds. The feature-laden output journeys onwards to the HL of the succeeding RBM, which instigates a cascading layer-wise training model. This final RBM incorporates its predecessor’s HL, thereby providing the seamless progression and continuity of extraction features through the network.

Eventually, the concluding RBM funnels the information handled into the topmost output layer. Now, the complex BP model takes the helm, which orchestrates the finetuning of the overall network. In the following, the prediction method is given:

The input vector X  is allocated to the $y_{1}$ VL and the following equation computes the probability that the neuron from the HL is turned on:

(16) $$P\left(H_{1}|V_{1}\right)=\sigma \left(W_{H_{1}}V_{1}+b_{1}\right)$$

In Equation (16), b shows the bias of VL, and σ indicates the sigmoid function.

(17) $$H_{1}∼P\left(H_{1}|V_{1}\right)$$

In Equation (17), $H_{1}$ is used to create the VL of the next RBM and compute the probability that the neuron from the VL is turned on:

(18) $$P\left(V_{2}|H_{1}\right)=\sigma \left(W_{V_{2}}H_{1}+a_{2}\right)$$

In Equation (18), a is the bias of HL, and after computing the probability density, the Gibbs sampling is carried out:

(19) $$V_{2}∼P\left(V_{2}|H_{1}\right)$$

Update weight and bias.

(20) $$\left\{\begin{array}{l} W\leftarrow W+lr[P\left(H_{1}|V_{1}\right)V_{1}^{T}-P\left(H_{2}|V_{2}\right)V_{2}^{T}]\\ b\leftarrow b+lr[V_{1}-V_{2}]\\ a\leftarrow a+lr[H_{1}-H_{2}] \end{array}\right.$$

In Equation (20), the learning rate can be represented as lr.

The BP algorithm is used to minimalize the objective function, viz., mean square error, and to finetune the weight of the network:

(21) $$E=\frac{1}{m}\sum_{i=1}^{m}(Y_{i}^{'}-Y_{i}{)}^{2}$$

In Equation (21), m represents the number of samples and $Y_{i}$ and $Y_{i}$ exemplify the actual and ideal outputs of the resultant layer.

## 4. Results and Discussion

**Figure 3:**
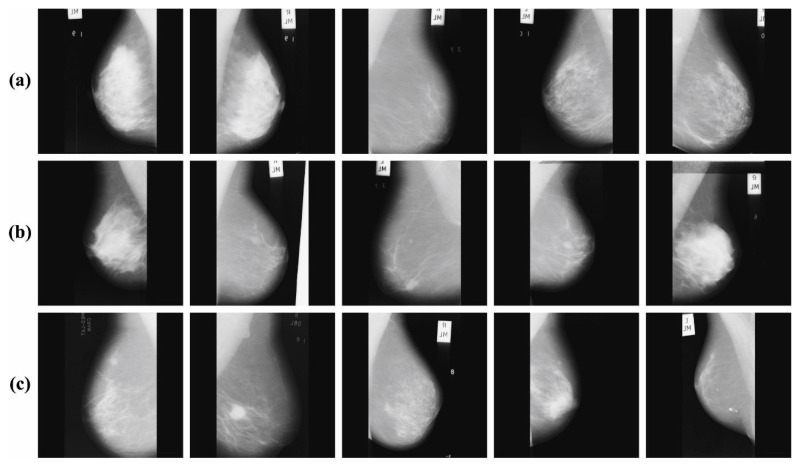
(**a**) Normal, (**b**) benign, (**c**) malignant.

In this section, the simulation analysis of the BMCA-AOADL methodology takes place using the MIAS dataset [27], comprising 1000 instances with three classes, as depicted in Table 1. Figure 3 exemplifies the sample images.

**Figure 4:**
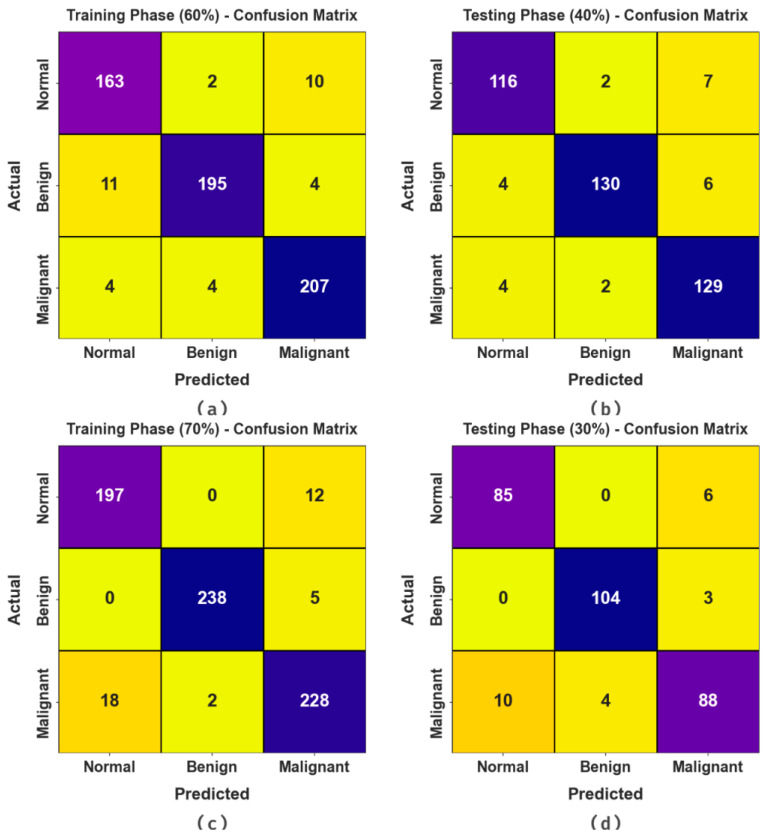
Confusion matrices of (**a**,**b**) 60:40 of TR set/TS set and (**c**,**d**) 70:30 of TR set/TS set.

Figure 4 reveals the confusion matrices achieved via the BMCA-AOADL method under 60:40 and 70:30 of the TR set/TS set. The outcome demonstrated the effective recognition and classification of three class labels.

**Figure 5:**
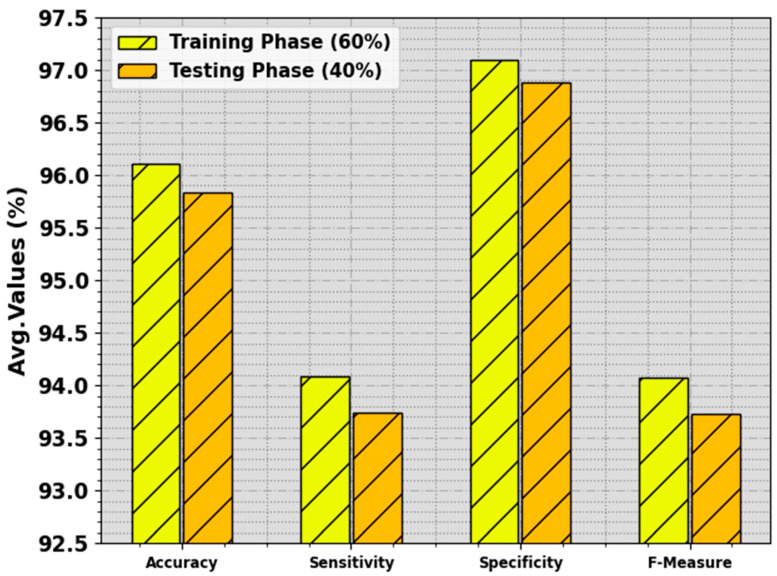
Average of BMCA-AOADL algorithm at 60:40 of TR set/TS set.

In Table 2 and Figure 5, the BC classification outcome of the BMCA-AOADL system is demonstrated with 60:40 of the TR set/TS set provided. The results indicate that the BMCA-AOADL technique effectually categorized three classes. On the 60% TR set, the BMCA-AOADL technique gained average $accu_{y}$, $sens_{y}$, $spec_{y}$, and $F_{measure}$ of 96.11%, 94.09%, 97.10%, and 94.07%, respectively. Moreover, on the 40% TS set, the BMCA-AOADL algorithm offered average $accu_{y}$, $sens_{y}$, $spec_{y}$, and $F_{measure}$ of 95.83%, 93.74%, 96.88%, and 93.73%, correspondingly.

**Figure 6:**
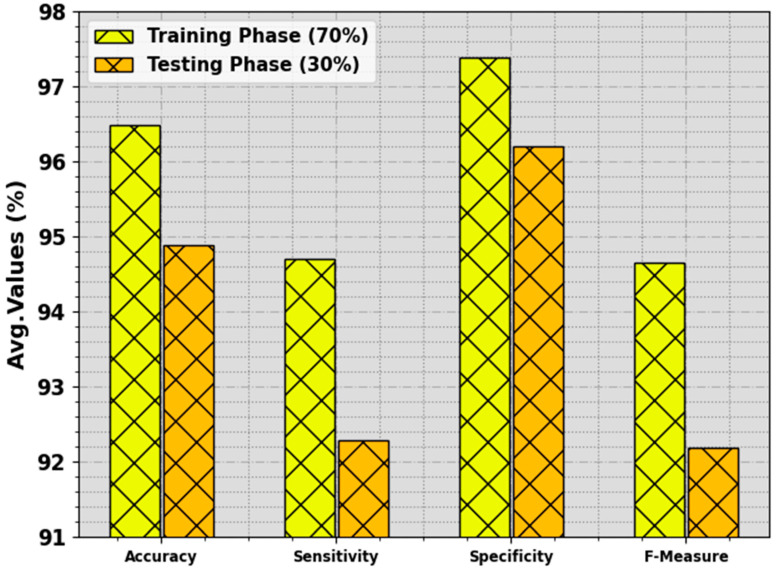
Average of BMCA-AOADL algorithm at 70:30 of TR set/TS set.

In Table 3 and Figure 6, the BC classifier outcome of the BMCA-AOADL algorithm is depicted with 70:30 of the TR set/TS set provided. The simulation value showed that the BMCA-AOADL system effectively categorized three classes. On the 70% TR set, the BMCA-AOADL system reached average $accu_{y}$, $sens_{y}$, $spec_{y}$, and $F_{measure}$ of 96.48%, 94.71%, 97.38%, and 94.66%, correspondingly. Furthermore, on the 30% TS set, the BMCA-AOADL methodology obtained average $accu_{y}$, $sens_{y}$, $spec_{y}$, and $F_{measure}$ of 94.89%, 92.29%, 96.20%, and 92.19%, correspondingly.

**Figure 7:**
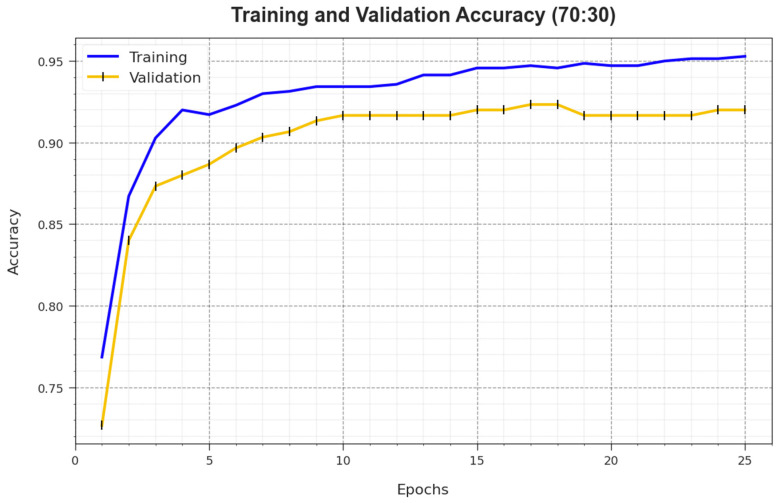
$Accu_{y}$ curve of BMCA-AOADL algorithm at 70:30 of TR set/TS set.

Figure 7 illustrates the training accuracy $TR\_accu_{y}$ and $VL\_accu_{y}$ of the BMCA-AOADL system at 70:30 of the TR set/TS set. The $TL\_accu_{y}$ is defined by the assessment of the BMCA-AOADL system on the TR dataset whereas the $VL\_accu_{y}$ is computed by estimating the performance on a separate testing dataset. The outcomes displayed that $TR\_accu_{y}$ and $VL\_accu_{y}$ caused an upsurge with an increase in epochs. Therefore, the performance of the BMCA-AOADL technique improved upon the TR and TS dataset with a rise in the number of epochs.

**Figure 8:**
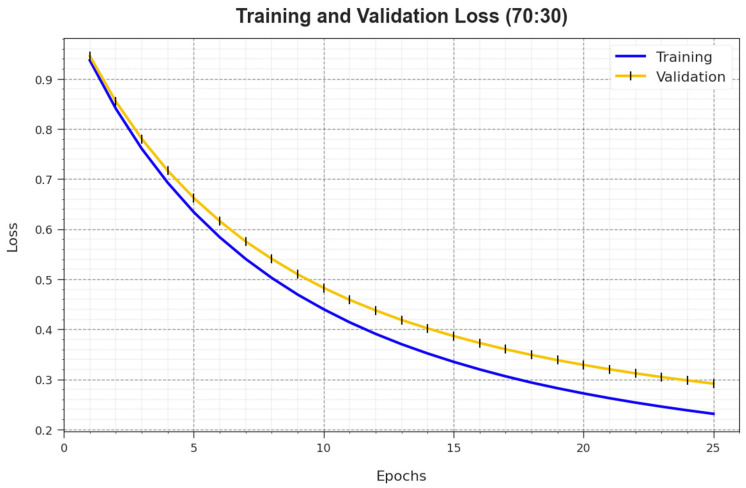
Loss curve of BMCA-AOADL algorithm at 70:30 of TR set/TS set.

In Figure 8, the TR_loss and VR_loss curves of the BMCA-AOADL system at 70:30 of the TR set/TS set are depicted. The TR_loss defines the error among the predictive solution and original values on the TR data. The VR_loss represents the measure of the performance of the BMCA-AOADL method on individual validation data. The outcomes show that the TR_loss and VR_loss generally decreased with rising epochs. This portrayed the enhanced performance of the BMCA-AOADL methodology and its ability to produce accurate classification. The lesser value of TR_loss and VR_loss demonstrated the enhanced performance of the BMCA-AOADL method in capturing patterns and relationships.

**Figure 9:**
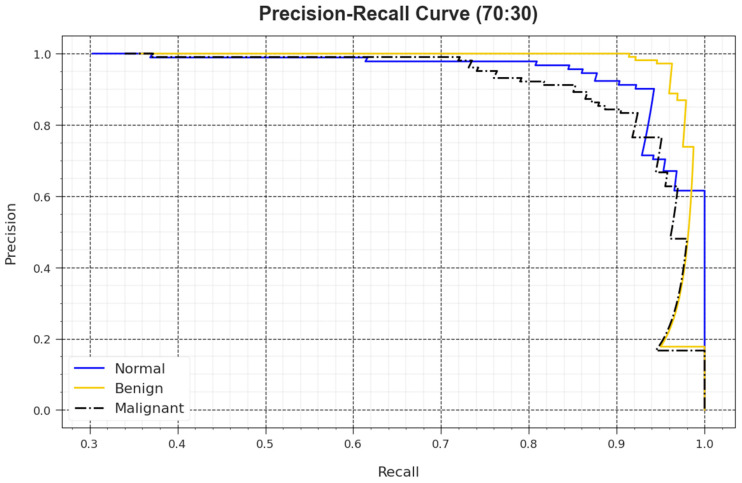
PR curve of BMCA-AOADL algorithm at 70:30 of TR set/TS set.

A detailed PR analysis of the BMCA-AOADL algorithm is defined at 70:30 of the TR set/TS set in Figure 9. The simulation values demonstrated that the BMCA-AOADL algorithm had better outcomes in enhancing PR values. Thus, it can be clear that the BMCA-AOADL algorithm attained greater performances of PR on three class labels.

**Figure 10:**
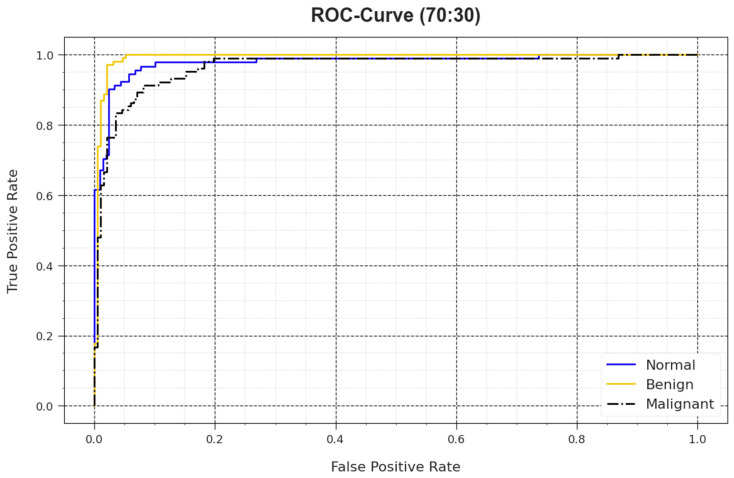
ROC curve of BMCA-AOADL algorithm at 70:30 of TR set/TS set.

In Figure 10, a ROC outcome of the BMCA-AOADL method is demonstrated at 70:30 of the TR set/TS set. The outcome value exposed that the BMCA-AOADL approach led to better values of ROC. Moreover, it was obvious that the BMCA-AOADL technique achieved superior values of ROC in three classes.

**Figure 11:**
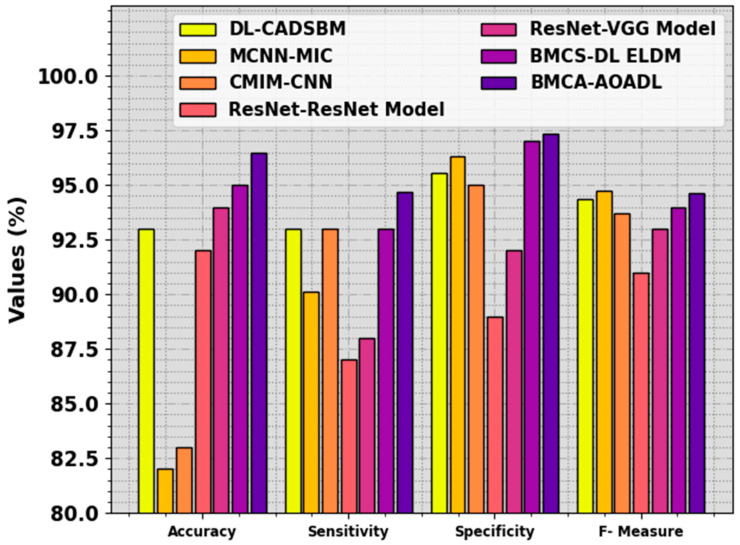
Comparative outcome of BMCA-AOADL approach with other DL methods.

The performance of the BMCA-AOADL technique is compared with recent DL models in Table 4 and Figure 11 [12]. The result stated that the MCNN-MIC and CMIM-CNN models showed the lowest performance. At the same time, the ResNet, ResNet-VGG, and BMCS-DL ELDM models illustrated moderately closer results. Additionally, the DL-CADSBM model reached considerable performance with $accu_{y}$, $sens_{y}$, $spec_{y}$, and $F_{measure}$ of 82%, 90.13%, 96.31%, and 94.76%, respectively. But the BMCA-AOADL technique surpassed the other ones with improved $accu_{y}$, $sens_{y}$, $spec_{y}$, and $F_{measure}$ of 96.48%, 94.71%, 97.38%, and 94.66%, respectively. Thus, the BMCA-AOADL technique can be applied to the automated breast mass classification process.

## 5. Conclusions

In this article, we presented an automated breast mass classification approach named the BMCA-AOADL technique on Digital Mammograms. The main intention of the BMCA-AOADL technique is to exploit the DL model with a bio-inspired algorithm for breast mass classification. In the presented BMCA-AOADL approach, the MF-based noise removal, U-Net segmentation, the SqueezeNet feature extractor, AOA-based hyperparameter tuning, and DBN classification are involved. For feature extraction, the BMCA-AOADL technique utilizes the SqueezeNet model with AOA as a hyperparameter tuning approach. To detect and classify the breast mass, the BMCA-AOADL technique applies the DBN algorithm. The simulation value of the BMCA-AOADL approach can be studied on the MIAS dataset. The experimental values displayed the significant outcomes of the BMCA-AOADL technique compared to other DL models with a maximum accuracy of 96.48%. Future work can perform comprehensive clinical validation and testing of the BMCA-AOADL approach on a large and diverse dataset of digital mammograms. This will help assess its real-world performance and generalizability. In addition, future work can enhance the interpretability and explainability of the BMCA-AOADL model’s predictions. This will help build trust among healthcare professionals and patients by providing insights into why a particular classification decision was made.

## Figures and Tables

**Table 1:** Details on the database.

Class	No. of Samples
Normal	300
Benign	350
Malignant	350
Total Samples	1000

**Table 2:** BC classifier outcome of BMCA-AOADL algorithm at 60:40 of TR set/TS set.

Class	$Accu_{y}$	$Sens_{y}$	$Spec_{y}$	$F_{Measure}$
**TR set (60%)**				
Normal	95.50	93.14	96.47	92.35
Benign	96.50	92.86	98.46	94.89
Malignant	96.33	96.28	96.36	94.95
Average	96.11	94.09	97.10	94.07
**TS set (40%)**				
Normal	95.75	92.80	97.09	93.17
Benign	96.50	92.86	98.46	94.89
Malignant	95.25	95.56	95.09	93.14
Average	95.83	93.74	96.88	93.73

**Table 3:** BC classifier outcome of BMCA-AOADL algorithm at 70:30 of TR set/TS set.

Class	$Accu_{y}$	$Sens_{y}$	$Spec_{y}$	$F_{Measure}$
**TR set (70%)**				
Normal	95.71	94.26	96.33	92.92
Benign	99.00	97.94	99.56	98.55
Malignant	94.71	91.94	96.24	92.49
Average	96.48	94.71	97.38	94.66
**TS set (30%)**				
Normal	94.67	93.41	95.22	91.40
Benign	97.67	97.20	97.93	96.74
Malignant	92.33	86.27	95.45	88.44
Average	94.89	92.29	96.20	92.19

**Table 4:** Comparative outcome of BMCA-AOADL approach with other DL methods [12].

Methods	$Accu_{y}$	$Sens_{y}$	$Spec_{y}$	$F_{Measure}$
DL-CADSBM	93.00	93.00	95.58	94.34
MCNN-MIC	82.00	90.13	96.31	94.76
CMIM-CNN	83.00	93.00	95.01	93.70
ResNet-ResNet	92.00	87.00	89.00	91.00
ResNet-VGG	94.00	88.00	92.00	93.00
BMCS-DL ELDM	95.00	93.00	97.00	94.00
BMCA-AOADL	96.48	94.71	97.38	94.66

## Data Availability

Data sharing does not apply to this article as no datasets were generated during the current study.
